# Asymmetric Requirement of Surface Epithelial *β-Catenin* During the Upper and Lower Jaw Development

**DOI:** 10.1002/dvdy.23755

**Published:** 2012-02-21

**Authors:** Ye Sun, Ian Teng, Randi Huo, Michael G Rosenfeld, Lorin E Olson, Xiaokun Li, Xue Li

**Affiliations:** 1 Department of Urology, Children's Hospital Boston, and Department of Surgery and Pathology, Harvard Medical SchoolBoston, and Harvard Stem Cell Institute, Cambridge, Massachusetts; 2 School of Pharmaceutical Science, Wenzhou Medical College, Wenzhou, and Norman Bethune College of Medicine, Jilin UniversityChangchun, China; 3 Howard Hughes Medical Institute, School of Medicine, University of California at San DiegoLa Jolla, California; 4 Immunobiology and Cancer Research Program, Oklahoma Medical Research FoundationOklahoma City, Oklahoma

**Keywords:** craniofacial, pharyngeal arch, jaw; Wnt, ß-catenin, Fgf8, Bmp4, Shh, endothelin, Dlx5, Hand2, Cre

## Abstract

Background: Intercellular communication between epithelial and mesenchymal cells is central to mammalian craniofacial development. β-*catenin* is the gateway of canonical *Wnt* signaling, one of the major evolutionarily conserved cell–cell communication pathways in metazoa. In this study, we report an unexpected stage- and tissue-specific function of β-*catenin* during mammalian jaw development. Results: Using a unique mouse genetic tool, we have discovered that epithelial β-*catenin* is essential for lower jaw formation, while attenuation of β-*catenin* is required for proper upper jaw development. Changes in β-*catenin* in vivo alter major epithelial *Fgf8*, *Bmp4*, *Shh*, and *Edn1* signals, resulting in partial transcriptional reprogramming of the neural crest-derived mesenchyme, the primary source of jawbones. Conclusions: The Wnt/β-*catenin* signal coordinates expression of multiple epithelial signals and has stage-specific asymmetric functions during mammalian upper and lower jaw development. In addition, these findings suggest that evolutionary changes of the canonical *Wnt/β-catenin* signaling pathway may lead to innovation of jaws. Developmental Dynamics 241:663–674, 2012. © 2012 Wiley Periodicals, Inc.

**Key Findings:**

- Mammalian epithelial Wnt/β-catenin signaling has asymetric functions in the upper and lower jaw development.
- The canonical Wnt/β-catenin pathway coordinates expression of multiple epithelial signals including *Fgf8*, *Bmp4*, *Shh*, and *Edn1*.
- Activation of epithelial Wnt/β-catenin signaling induces molecular transformation of the upper jaw to the lower jaw mesenchymal phenotype.
- Evolutionary changes of the canonical *Wnt/β-catenin* signaling pathway may lead to innovation of jaws.

## INTRODUCTION

Exquisite vertebrate facial structures are results of gradual transformations of relatively simple embryonic mesenchymal swellings or prominences, including βthe first pharyngeal arch (PA1; Helms and Schneider,^2003^; Santagati and Rijli,^2003^; Kuratani,^2005^; Depew and Simpson,^2006^). The dorsal maxillary (mx) and ventral mandibular (md) components of the PA1 are the principal sources of upper jaw and lower jaw structures, respectively. Although mesenchymal cells of the PA1 prominences have intrinsic jaw identity (Schneider and Helms,^2003^), growth and patterning of pharyngeal mesenchyme depends on molecular cues from the surrounding epithelial cells. For instance, the foregut endoderm and the frontonasal ectoderm play instructive roles in specification of lower and upper beak, respectively (Couly et al.,^2002^; Hu et al.,^2003^). The molecular basis underlying epithelial and mesenchymal cell interactions during jaw specification has yet to be fully elucidated.

The *Wnt/β-catenin* signaling pathway is one of the most ancient intercellular communication pathways, in which intracellular β-*catenin* acts as a central node to transduce canonical *Wnt* signals (Richards and Degnan,^2009^; Pang et al.,^2010^). Canonical Wnt signaling stabilizes and enhances β-*catenin* function as a transcription coactivator to regulate downstream target gene expression. The *Wnt/β-catenin* pathway has multiple roles including establishing primary body axis, formation of individual organs, and maintenance of tissue homeostasis. One of the conserved functions of this pathway is to convey positional information to promote posterior or caudal identity (Martin and Kimelman,^2009^; Petersen and Reddien,^2009^). This function can be traced to prebilaterians, the basal metazoans that diverged from bilaterians over 500 million years ago (Hobmayer et al.,^2000^; Wikramanayake et al.,^2003^; Kusserow et al.,^2005^; Pang et al.,^2010^).

Genetic analyses of mouse β-*catenin* have uncovered diverse functions such that the level and timing of β-catenin activity triggers different and sometimes opposing biological outcomes (Grigoryan et al.,^2008^). Studies in chick and mouse have shown that levels of the *Wnt/β-catenin* signaling correlate with region-specific growth and fusion of craniofacial prominences, suggesting that changes in *Wnt/β-catenin* activity may underlie evolutionary adaptations and variations of species-specific facial appearances, such as chick beak and mouse muzzle (Brugmann et al.,^2007^). *Wnt/β-catenin* signaling induces neural crest cell formation (Garcia-Castro et al.,^2002^), and is required for survival of PA1 mesenchymes during craniofacial morphogenesis (Brault et al.,^2001^; Reid et al.,^2011^; Wang et al.,2011b). In this study, we examined surface epithelial-specific functions of β-*catenin* in the PA1 development. Our findings demonstrate that β-*catenin* is a key regulator of multiple epithelial signals and has distinct functions in the patterning upper and lower jaws.

## RESULTS

### Epithelial *β-catenin* Is Essential for Craniofacial Morphogenesis

**Fig. 1:**
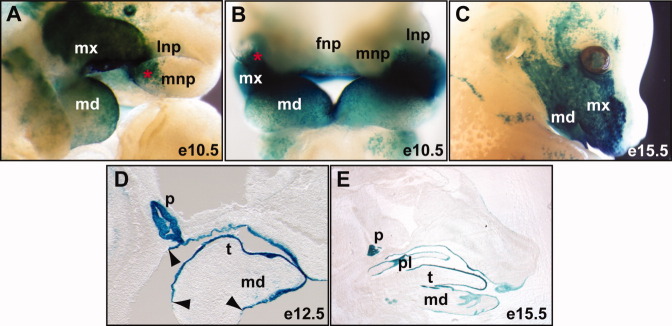
Epithelial-specific *Pitx1-Cre* transgenic mice. X-gal staining (blue) of Cre activities of the *Pitx1/Cre* and *R26R*^*lacZ*^ double heterozygous embryos. **A–C:** Whole-mount staining. **D,E:** Middle sagittal sections. Arrowheads point to the caudal limits of Cre activity; asterisk, ventral nasal placode; fnp, frontal nasal prominence; lnp, lateral nasal prominence; md, mandible prominence; mnp, medial nasal prominence; mx, madillary prominence; p, pituitary; pl, palate; t, tongue.

A *Cre* transgenic line driven by a *Pitx1* enhancer, *Pitx1/Cre*, is expressed in the oral epithelium but not the pharyngeal mesenchymes (Olson et al.,^2006^) Using a *R26R*^*lacZ*^ reporter line (Soriano,^1999^), we demonstrated its epithelial-specific Cre activity in the PA1 (Fig. 1). Whole-mount X-gal staining of embryonic day (E) 10.5 embryos demonstrated strong Cre activity in the surface epithelium of the PA1, including both ventral mandibular (mdPA1) and dorsal maxillary (mxPA1) components, and the oral epithelium (Fig. 1). The X-gal-positive cells extended dorsally and rostrally to the optic region and to the ventral third of the nasal placode, respectively. The caudal limits reached to the second PA. Sagittal sections of E12.5 embryos demonstrated that the entire oral epithelium was stained with X-gal (Fig. 1D). Sharp caudal boundaries of the oral epithelium were found near pituitary gland and tongue primordium (Fig. 1D), demonstrating that Cre activity was restricted to ectoderm lineages. At E15.5, strong X-gal staining was found in surface epithelial cells covering the mandible, maxilla, and to a lesser extent, the optic and external ear regions (Fig. 1C,E).

**Fig. 2:**
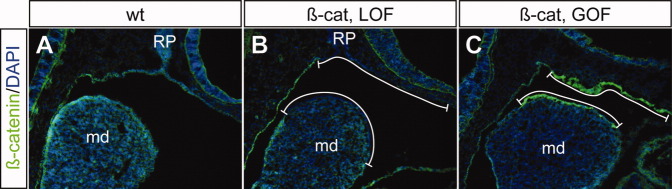
Conditional manipulation of epithelial β-*catenin*. **A–C:** Immunohistochemical analyses of sagittal cryostat sections from E10.5 wild-type (A, wt) and β-*catenin* loss-of-function (B, LOF) and gain-of-function (C, GOF) mutants using β-catenin-specific antibody. bracket, epithelial subdomain where epithelial β-*catenin* is conditionally altered; md, mandibular; RP, Rathke's pouch.

**Fig. 3:**
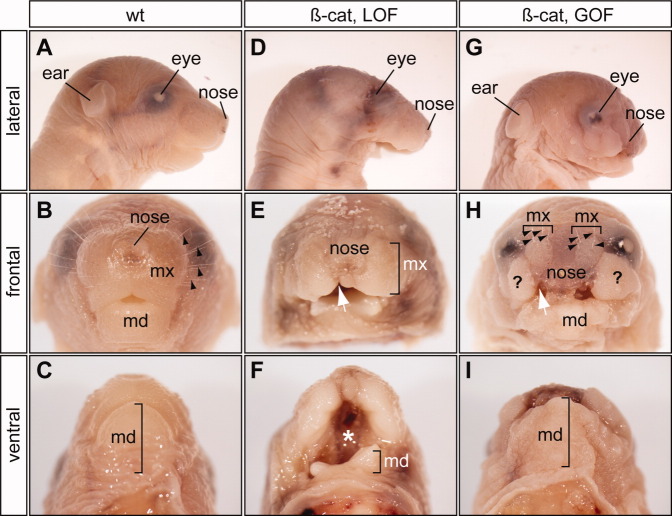
Asymetric requirements for epithelial β-*catenin* in upper and lower jaw formation. **D–F:** Newborn lower jaw defects of epithelial-specific β-*catenin* loss-of-function mutants (β-cat, LOF), and upper jaw defects of the gain-of-function mutants (β-cat, GOF, **G-I**). **A–C:** Wild-type controls. White arrow, cleft lips; black arrow, whiskers; asterisk, cleft palate; md, mandible; mx, maxillary; question mark, ectopic soft tissue.

**Fig. 4:**
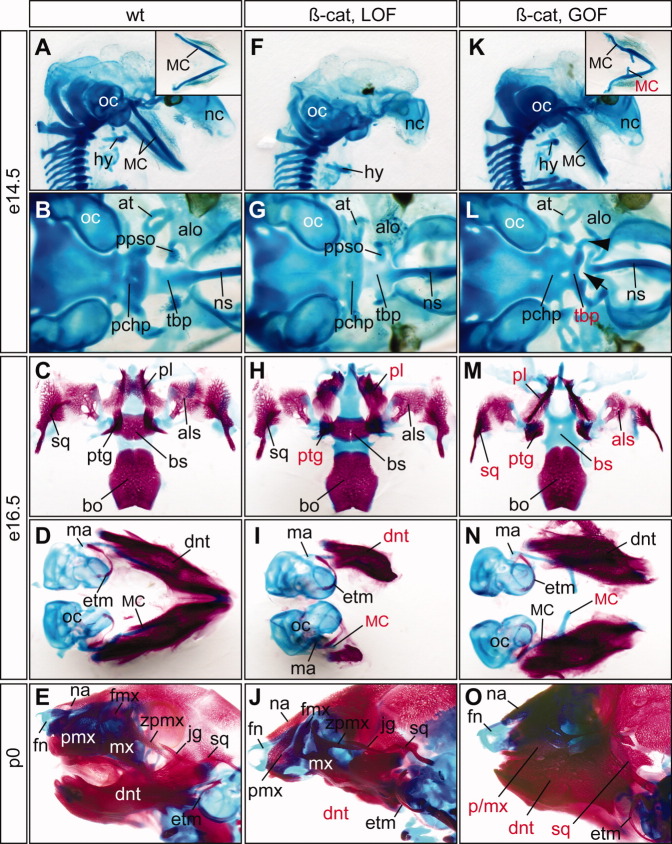
Cartilage and bone defects of epithelial-specific β-*catenin* mutants. A–O: Alcian blue cartilage staing of of embryonic day (E) 14.5 (A,B,F,G,K,L), E16.5 embryos (C,D,H,I,M,N), and newborn pups (E,J,O) and alizarin red bone staining of E16.5 embryos and newborn pups. **A–E:** Wild-type controls. **F–J:** β-*cat*^*eLOF*^ mutants display severe truncation of Meckel's cartilage (MC) and dentary bone, deformation of palatine (pl) and pterygoid (ptg). **K–O:** β-*cat*^*eGOF*^ mutants have ectopic MC and upper jaw defects. Red labels indicate defective structures. All pictures are ventral views except A, E, F, J, K and O, which are sagittal views. Inserts in A and K show ventral view of dissected MC. Note that the dentary bones of the β-*cat*^*eGOF*^ (N) are connected distally but dissociated during staining and dissection process. Arrow, gap; arrowhead, unidentified cartilage rod; at, ala temporalis; alo, ala orbitalis; als, alisphenoid; bo, basioccipital; bs, basisphenoid; dnt, dentary; etm, ectotympanic; fmx, frontal process of maxilla; fn, frontal nasal cartilage; hy, hyoid; jg, jugal; ma, malleus; mx, maxilla; mc, Meckel's cartilage; na, nasal bone; nc, nasal capsule; ns, nasal septum; oc, otic capsule; pchp, parachordal plate; pl, palatine; ppso, pila postoptica; ptg, pterygoid; sq, squamosal; tbp, trabecular basal plate; zpmx, zygomatic process of maxilla.

To determine potential roles of epithelial *Wnt/β-catenin* signaling during craniofacial development, we combined *Pitx1/Cre* with two different β-*catenin* alleles that permit conditional deletion of the β-*catenin* gene (β-*cat*^*ex2-6*^; Brault et al.,^2001^) or stabilization of β-catenin protein (β-*cat*^*ex3*^; Harada et al.,^1999^). We first confirmed changes of β-catenin levels in *Pitx1/Cre;β-Cat*^*ex2-6/ex2-6*^ and *Pitx1/Cre;β-Cat*^*ex3/+*^ embryos, the PA1 epithelial-specific β-*catenin* LOF (β-*cat*^*eLOF*^) and β-*catenin* GOF (β-*cat*^*eGOF*^) mutants respectively (Fig. 2). Immunohistochemical staining using β-catenin-specific antibody indicated high levels of β-catenin in the PA1 epithelium and Rathke's pouch in wild-type controls at E10.5. As expected, β-catenin was undetectable in β-*cat*^*eLOF*^ mutants, while it was markly increased in the β-*cat*^*eGOF*^ mutants. β-*Catenin* expression was unaffected in regions where Cre was not expressed. We next examined gross defects of β-*cat*^*eLOF*^ and β-*cat*^e*GOF*^ mutants (Fig. 3). All β-*cat*^*eLOF*^ and β-*cat*^*eGOF*^ mutants survived to birth but exhibited severe craniofacial defects (Fig. 3). The mandibles of β-*cat*^*eLOF*^ mutants were absent (Fig. 3D–F), but were present in β-*cat*^*eGOF*^ mutants (Fig. 3G–I). Conversely, the maxillas of β-*cat*^*eGOF*^ mutants were severely deformed (Fig. 3G–I). The area of maxillary soft tissue and whisker pads was much smaller in β-*cat*^*eGOF*^ mutants than in wild-type controls and was displaced dorsally by an unidentified soft tissue mass (Fig. 3H). The frontal nasal structures in both mutants were hypoplastic (Fig. 3), but nasal capsule cartilage was not affected (Fig. 4). Consistent with the essential role of epithelial β-*catenin* function during the second palate formation (He et al.,^2011^), both mutants had cleft lip and palate defects (Fig. 3F and data not shown). Together, these gross phenotypes demonstrated the important role of epithelial β-*catenin* during craniofacial development.

### Asymetric Requirements for Epithelial *β-catenin* in Upper and Lower Jaw Formation

The phenotypic differences of β-*cat*^*eLOF*^ and β-*cat*^*eGOF*^ mutants is intriguing because it suggests that epithelial β-*catenin* has a differential impact on upper and lower jaw formation (Fig. 3). To further examine whether epithelial β-*catenin* regulates formation of the neural crest-derived skeletal structures, we analyzed craniofacial cartilages and bones of β-*cat*^*eLOF*^ and β-*cat*^*eGOF*^ mutants (Fig. 4). Overall, the frontal nasal cartilages from both LOF (Fig. 4F,J) and GOF (Fig. 4K,O) mutants were intact. Formation of the upper and lower jaw skeletal structures, however, exhibited distinct sensitivities to altered levels of epithelial β-*catenin* (Fig. 4).

The β-*cat*^*eLOF*^ mutants exhibited severe distal truncation of the mdPA1-derived lower jaw Meckel's cartilage at E14.5 (Fig. 4F). The most ventral mdPA1-derived structures, including the rostral process, lower incisor, and distal dentary bones, were all missing from the E16.5 embryos and the newborn pups (Fig. 4H–J). The dorsal mdPA1-derived bones, including the ectotympanic ring, gonial, and malleus, were not affected (Fig. 4I,J). Consistent with the cleft palate phenotype, the palatine and pterygoid bones were deformed in β-*cat*^*eLOF*^ mutants (Fig. 4H). However, other mxPA1-derived skeletal elements, including maxilla, jugal, lamina obturans, squamosal, incus, and the associated skeletal structures including parachordal and trabecular cartilage plates, and basioccipital and basisphenoid bones, were normal (Fig. 4J). These findings demonstrate that formation of the lower jaw, but not the upper jaw, is particularly sensitive to reduced levels of epithelial β-*catenin*.

Complementary to the agenesis of the Meckel's cartilage of the LOF mutants, β-*cat*^*eGOF*^ mutants formed ectopic Meckel's cartilage at E14.5 and E16.5 (Fig. 4K,N). These ectopic cartilage were formed at the proximal region of the bent Meckel's cartilage rods at E14.5, and the dentary bones were deformed at E16.5 (Fig. 4K,N). The resulting lower jawbones were enlarged and fused with the putative upper jawbones of the newborn β-*cat*^*eGOF*^ mutants (Fig. 4O). The mxPA1-derived upper jaw structures, including alisphenoid and squamosal bones, were underdeveloped (Fig. 4M), and were completely fused with dentary bones and severely deformed in the newborn pups (Fig. 4O). The premaxilla and maxilla were also fused with the dentary bones. The jugal bone was missing. In addition, the trabecular plate failed to connect to the nasal septum (Fig. 4L). A conspicuous rod shaped cartilage structure, most likely the deformed cartilage of the postoptic pillar of optic capsule, extended bilaterally from rostral end of the trabecular plate toward the ala orbitalis cartilage (Fig. 4L, arrowhead). The basisphenoid bone was missing in β-*cat*^*eGOF*^ mutants (Fig. 4M). The palatine bones failed to meet at the midline and the pterygoid bones were severely deformed (Fig. 4M). Thus, in contrast to the LOF mutants, stabilization of epithelial β-catenin protein in the GOF mutants resulted in severe defects of the mxPA1-derived skeletal structures.

Collectively, facial skeletal analyses of both β-*catenin* LOF and GOF mutants suggested that epithelial β-*catenin* is required for formation of the lower jawbones while attenuation of epithelial β-*catenin* is necessary for proper upper jawbone development.

### *β-catenin* Is Genetically Upstream of Multiple Epithelial Signals

**Fig. 5:**
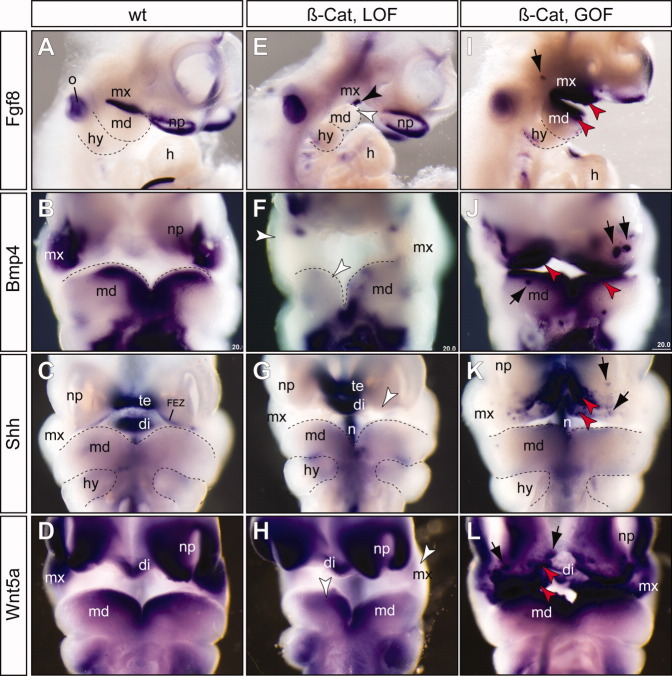
β-*catenin* functions upstream of other epithelial signals. **E–L:** RNA in situ hybridization (purple color) of embryonic day (E) 10.5 embryos using gene-specific probes indicates decreased expression of *Fgf8*, *Bmp4*, *Shh*, and *Wnt5a* in the β-*catenin* loss-of-function (β-*cat*, LOF) mutants (E–H), while expression of these genes were enhanced in the β-*catenin* gain-of-function (β-*cat*, GOF) mutants (I–L). **A–D:** Wild-type controls. **I–L:** Patchy ectopic staining (black arrows) of *Fgf8* (I), *Bmp4* (J), *Shh* (K), and *Wnt5a* (L) were observed in the β-*catenin* GOF mutants. All pictures are frontal views except A, E and I, which are lateral views. di, diencephalon; FEZ, frontonasal ectoderm zone; h, heart; hy, hyoid arch; md, mandibular prominence; mx, maxillary prominence; n, notochord/floor plate; np, nasal placode; o, otic vesicle; te, telencephalon; black arrowhead, residual expression of *Fgf8* at the cleft of between mx and md; white arrowhead, reduced expression; red arrowhead, enhanced expression.

**Fig. 6:**
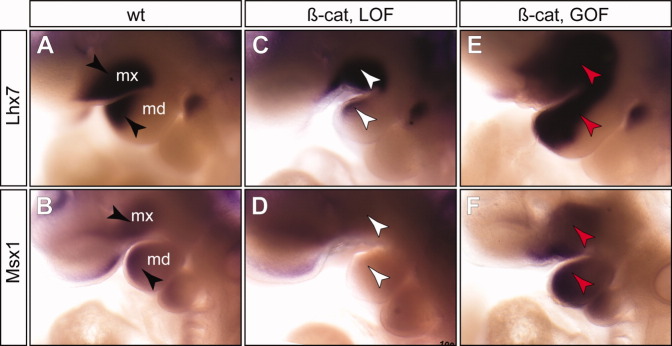
Epithelial β-*catenin* indirectly regulates expression of genes in the mesenchymal cells. Whole-mount RNA in situ hybridization using *Lhx7* and *Msx1* probes. **A,B:** Wild-type controls. **C,D:** Epithelial-specific β-*catenin* loss-of-function (β-*cat*, LOF) mutants. **E,F:** β-*catenin* gain-of-function (β-*cat*, GOF) mutants. black arrowhead, normal expression pattern; white arrowhead, reduced expression; red arrowhead, increased expression.

To determine whether changes in epithelial β-*catenin* caused alterations in epithelial signals with consequent changes of mesenchymal cell patterning, we analyzed expression of *Fgf8* and *Bmp4* (Fig. 5). *Fgf8* was expressed in PA epithelium of wild-type control embryos at E10.5 (Fig. 5A). Previous studies demonstrated that conditional inactivation of *Fgf8* at E9.0 resulted in truncation of the proximal and intermediate regions of both mxPA1 and mdPA1 (Trumpp et al.,^1999^). In all β-*cat*^*eLOF*^ mutants analyzed (n = 5), *Fgf8* expression in the PA1 was reduced to a small patch of cells at the cleft region between mxPA1 and mdPA1 (Fig. 5E). A downstream transcriptional target of *Fgf8*, *Lhx7* (Trumpp et al.,^1999^), was concordantly reduced (Fig. 5C), confirming that epithelial *Fgf8* signaling activity was reduced. Complementary to the observations of β-*cat*^*eLOF*^ mutants, we found that *Fgf8* and *Lhx7* expression levels were increased and their expression domains were expanded in the β-*cat*^*eGOF*^ mutants (Figs. 5I, 6E).

Conditional deletion of *Bmp4* results in reduction of *Msx1/Msx2* expression and severe defects of the jawbones (Liu et al.,^2005^). While expression of *Bmp4* is independent of *Fgf8* (Trumpp et al.,^1999^), *Bmp4* acts an activator of *Fgf8* in the proximal mdPA1, and a repressor of *Fgf8* in the distal mdPA1 (Liu et al.,^2005^). In wild-type arches, we detected *Bmp4* at the distal ends of maxillary and mandibular epithelia of the PA1 (Fig. 5B). In β-*cat*^*eLOF*^ mutants, *Bmp4* was largely undetectable, and only small patches of cells at the most distal ends exhibited weak expression (Fig. 5F). Expression of a *Bmp4* downstream target gene, *Msx1*, was not detected in β-*cat*^*eLOF*^ mutants, consistent with the deficiency of *Bmp4* signaling in the mutants (Fig. 6D). In β-*cat*^*eGOF*^ mutants, we observed enhanced and expanded expression of *Bmp4* in epithelial cells and *Msx1* in mesechymal cells (Figs. 5J, 6F). Thus, we observed concerted shifts of epithelial *Bmp4* signal and its downstream target in mesenchymal cells, *Msx1*. Together, these results demonstrate that β-*catenin* is genetically upstream of critical epithelia signals including *Fgf8* and *Bmp4* during mammalian jaw development.

We also examined expression patterns of *Shh* and *Wnt5a* (Fig. 5). The *Shh*-positive frontal ectodermal zone and foregut endoderm are critical for craniofacial development (Couly et al.,^2002^; Hu et al.,^2003^; Brito et al.,^2006^; Hu and Marcucio,^2009^). In addition, *Shh* signaling controls reciprical epithelial-mesenchymal interactions in the outgrowth of the facial primordia (Jeong et al.,^2004^; Young et al.,^2010^), and the secondary palate development (Lan and Jiang,^2009^). In β-*cat*^*eLOF*^ mutants, *Shh* expression in the frontal ectodermal zone was undetectable, while expression in internal control regions including telencephalon, diencephalon and pharyngeal endoderm, were not affected (Fig. 5G). Previous studies in the chick have shown that *Shh*-expressing cells can induce ectopic supernumerary jaws (Brito et al.,^2008^). We found that in β-*cat*^*eGOF*^ mutants, *Shh* expression was enhanced at the frontal ectodermal zone and ectopically expressed at the distal mxPA1 and frontonasal regions, and to a lesser extent, the mdPA1 (Fig. 5K). Similar to *Bmp4* (Fig. 5J), ectopic *Shh* expression was often patchy in the frontal nasal region (Fig. 5K). *Wnt5a* is critical for outgrowth of the craniofacial prominences including the mdPA1 and mxPA1 (Yamaguchi et al.,^1999^). We found that *Wnt5a* was down regulated in β-*cat*^*eLOF*^ mutants (Fig. 5H), and increased and ectopically expressed in β-*cat*^*eGOF*^ mutants (Fig. 5L). Collectively, these findings demonstrated that epithelial β-*catenin* is an upstream regulator of multiple critical epithelial signals including *Fgf8*, *Bmp4*, *Shh*, and *Wnt5a*.

### Epithelial *β-catenin* Induces Molecular Reprogramming of Mesenchymal Cells

**Fig. 7:**
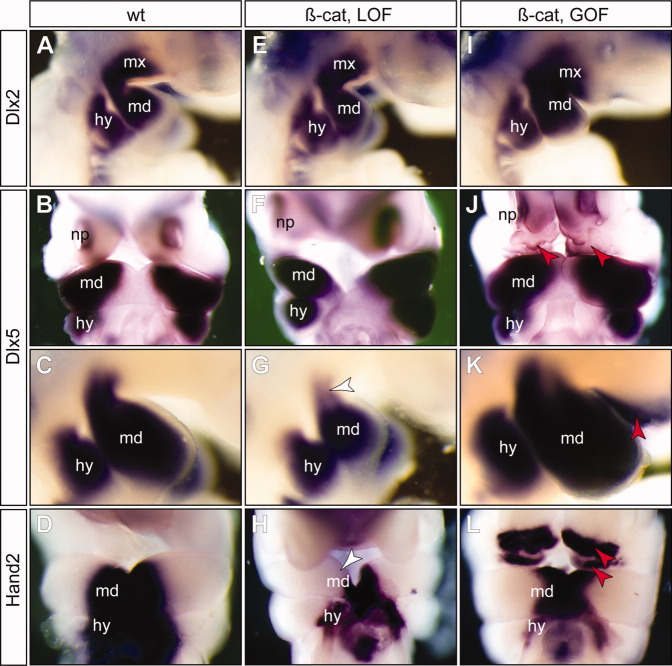
**A–L**: β-*catenin* induces partial reprogramming of maxillary mesenchyme. While expression of *Dlx2* is maintained in both β-*catenin* loss-of-function (β-*cat*, LOF) mutants (E) and gain-of-function (β-*cat*, GOF) mutants (I), expression of the mdPA1 genes *Dlx5* (B,C,F,G,J,K) and *Hand2* (D,H,L) are reduced in the LOF mutants (white arrowheads in G and H), and ectopically induced in the GOF mutants (red arrowheads in K and L). A,E,I,C,G,K: Lateral views and the rest are frontal views. See Figure 4 for abbreviations.

**Fig. 8:**
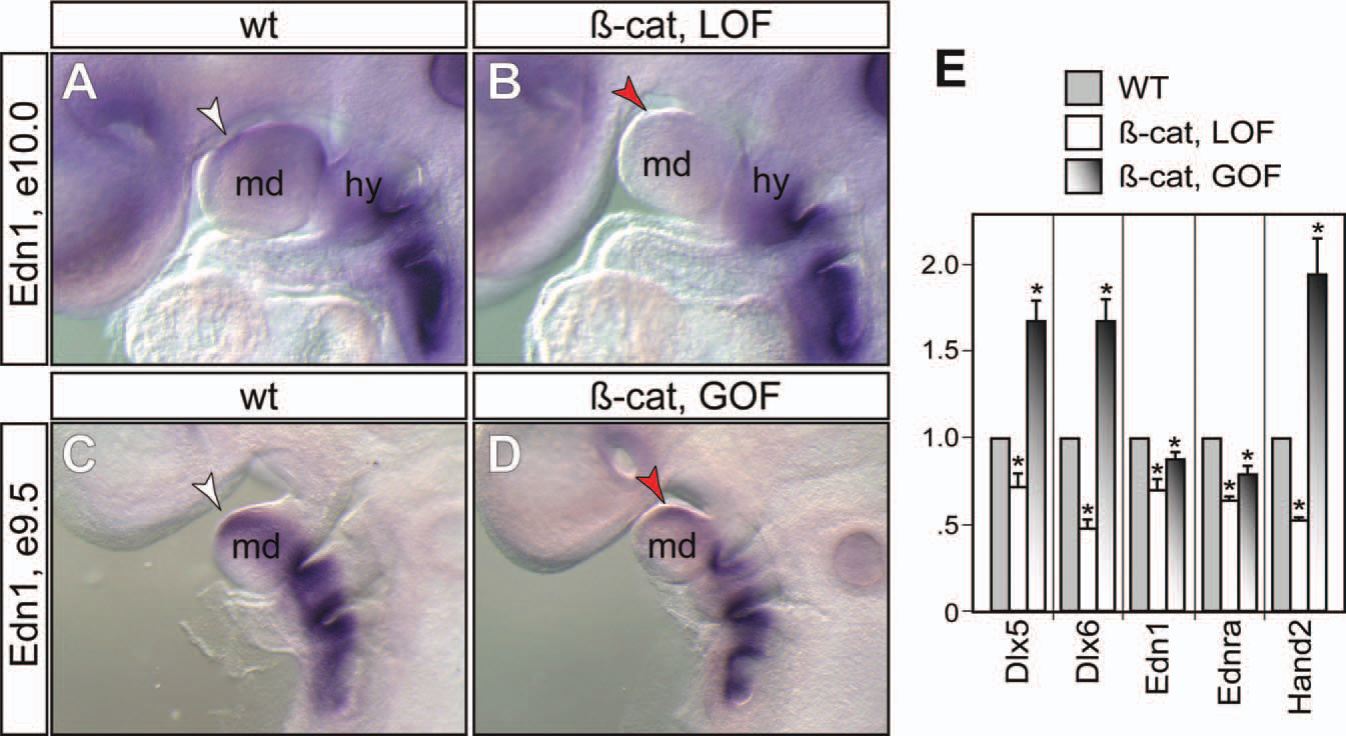
Expression of *Edn1* depends on epithelial β-*catenin*. **A–D:** Whole-mount RNA in situ hybridization using *Edn1* specific probe. white arrowhead, normal expression in mandibular prominence (md); red arrowhead, reduced expression in mutants. expression in hyoid arch (hy) and the rest of caudal arches are unaffected. **E:** Real-time quantitative polymerase chain reaction analyses of relative expression levels of genes (x-axis) in the microdissected first pharyngeal arch at embryonic day (E) 10.5 using β-*actin* as an internal control. Asterisk, *P* < 0.05 (y-axis), Student *t*-test, n = 4.

To determine whether altered epithelial β-*catenin* had the potential to alter mesenchymal cell differentiation programs, we examined expression patterns of transcription factors involved in the PA1 specification (Minoux and Rijli,^2010^; Fig. 7). *Dlx*-family homeodomain transcription factors are critical regulators of the PA1 (Depew et al.,^2005^; Jeong et al.,^2008^). *Dlx1* and *2* are expressed in both mdPA1 and mxPA1, whereas *Dlx5* and *6* are restricted to the mdPA1. Inactivation of *Dlx5* and *Dlx6* results in homeotic transformation of the lower jaw into the upper jaw structures (Beverdam et al.,^2002^; Depew et al.,^2002^). In our experiments, expression of *Dlx2* appeared to be independent of epithelial β-*catenin* as both LOF and GOF mutants had similar levels of *Dlx2* when compared with the wild-type littermate controls (Fig. 7A,D,G). However, *Dlx5* expression was slightly reduced at the dorsal mdPA1 region of β-*cat*^*eLOF*^ mutants (Fig. 7G, white arrowhead, and Fig. 8F) and ectopically expressed in β-*cat*^*eGOF*^ mutants at the frontal nasal region (Fig. 7J, red arrowheads, and 8F) and the oral region of mxPA1 (Fig. 7K, red arrowhead, and Fig. 8F).

We also examined the expression pattern of *Hand2*, which is an effector of *Dlx5* and *Dlx6* transcription factors (Charite et al.,^2001^; Sato et al.,^2008^). *Hand2* was reduced in the mdPA1 of β-*cat*^*eLOF*^ mutants (Figs. 7H, 8F). Furthermore, strong ectopic *Hand2*-positive cells were found at the frontal nasal and oral regions of the mxPA1 of β-*cat*^*eGOF*^ mutants (Fig. 7L, red arrowheads, and 8F). Conversion of the maxillary into a mandibular fate can occur through over expression of *Hand2* (Sato et al.,^2008^). Local ectopic activation of *Dlx5* and *Hand2* in β-*cat*^*eGOF*^ mutants therefore suggests that high levels of epithelial β-*catenin* can induce molecular reprogramming of mesenchymal cells from a maxillary to mandibular fate. Because *Dlx5/6* and *Hand2* are downstream effectors of the Endothelin-1 (*Edn1*)/Endothelin receptor type-A (*Ednra*) signaling pathway, and constitutive activation of *Edn1/Ednra* is sufficient to transform maxillary to mandibular identity (Sato et al.,^2008^), we therefore examined whether expressions of *Edn1* and *Ednra* were also affected by alteration of epithelial β-*catenin* levels (Fig. 8). Both *Edn1* and *Ednra* were significantly reduced in the mandibular region of β-*cat*^*eLOF*^ mutants, while their expressions were unaffected in the caudal arch regions where β-*catenin* expression was not perturbed (Fig. 8 and data not shown). Surprisingly, their expressions were also down-regulated, but to a lesser extent in β-*cat*^*eGOF*^ mutants. These molecular changes are consistent with the gross phenotypic observations that loss of β-catenin results in a lower jaw agenesis phenotype while overexpression of β-*catenin* is not sufficient to fully transform the first arch identity.

## DISCUSSION

Cell–cell communications are a quintessential feature of multicellular organisms and are, therefore, under tight evolutionary constraint. Key signaling pathways, including *Wnt*, *TGF*-β, *Hedgehog*, and *Notch*, were already present in the last common ancestors to all living bilaterians (Richards and Degnan,^2009^). Accordingly, variation and redeployment of these signaling pathways is likely to mediate the evolution of novel features such as evolution of beak shapes of Darwin's finches (Abzhanov et al.,^2004^) and acquisition of butterfly eyespots (Keys et al.,^1999^).

In this study, we demonstrated that epithelial β-*catenin* coordinates expression of several critical signaling molecules required for vertebrate jaw development. We found that high levels of epithelial β-*catenin* support lower jaw development while inducing molecular reprogramming of mesenchymal cells from a maxillary to mandibular-like fate. Deletion of epithelial β-*catenin* resulted in severe truncation or absence of the ventral mdPA1-derived skeletal structures, particularly the dentary bone, while stabilization of epithelial β-*catenin* enhanced dentary bone formation (Figs. 3, 4). These observations suggest that epithelial β-*catenin* activities have gradient effects controlling dorsoventral patterning of the PA1 and formation of jawbones. While epithelial β-*catenin* is essential for lower jaw formation, attenuation of its activities at later stages seems to be a prerequisite for normal development of the upper jaw. These surprising findings demonstrate that epithelial β-*catenin* has asymmetric functions during vertebrate jaw development: high levels promote lower jaw formation while low levels are required for upper jaw development. Changing levels of *Wnt/β-catenin* signaling have been implicated in the evolutionary adaptations and variations of species-specific facial appearances (Brugmann et al.,^2007^; Mallarino et al.,^2011^). Our studies support a hypothesis that changes of *Wnt/β-catenin* signaling were involved in vertebrate jaw evolution.

Different aspects of epithelial β-*catenin* functions in craniofacial development at earlier developmental stages were previously reported using a similar genetic strategy but with different *Cre*-expressing mouse lines. Wang et al. used a *Foxg1-Cre* knockin line, which is active at the anterior neural ridge, neural epithelia and adjacent frontal nasal ectoderm at E8.75 (Wang et al.,2011b). In contrast to our findings, LOF β-*catenin* mutants using *Foxg1-Cre* line resulted in agenesis of the mxBA1-derived maxillary structures due to anterior neural ridge defect at early developmental stages (Wang et al.,2011b). Williams and colleagues used a transgenic line that appeared to have an even earlier and broader Cre activity including frontal nasal ectoderm and pharyngeal ectoderm at E8.5 (Reid et al.,^2011^). A LOF in their studies resulted in agenesis of both mxPA1 and mdPA1 and a GOF resulted in early lethal phenotype. Unlike these *Cre* lines, the *Pitx1/Cre* line used in our study is restricted to the pharyngeal arch epithelium, but not in the frontal nasal epithelium. Furthermore, the *Pitx1/Cre* transgene has a later onset of *Cre* activity at E9.0 (Olson et al.,^2006^). The unique spatiotemporal properties of the *Pitx1/Cre* line enable us to uncover a previously unknown asymmetric function of epithelial β-*catenin* during patterning of upper and lower jaw structures. Taken together, these studies suggest that development of the lower jaw depends on continuous epithelial β-*catenin* activity while temporal early activation and late attenuation of epithelial β-*catenin* is required for normal upper jaw development.

Our studies indicate that epithelial β-*catenin* controls expression, either directly or indirectly, of other epithelial signaling molecules including *Fgf8*, *Bmp4*, *Shh*, *Wnt5a*, and *Edn1* (Figs. 5, 8). Concerted changes of these epithelial signals are likely responsible for mesenchymal cell proliferation and survival. Mesenchymal cell phenotypes may also influence the behavior of epithelial cells (Sheehy et al.,^2010^). Consistent with the notion that epithelial β-*catenin* might be involved in the specification of the PA1, a small portion of distal mxPA1 mesenchyme in β-*cat*^*eGOF*^ mutants assumed mdPA1-like molecular features, including expression of *Dlx5* and *Hand2* genes (Beverdam et al.,^2002^; Depew et al.,^2002^; Sato et al.,^2008^; Fig. 7H,I). Recent studies indicate that *Notch* antagonizes β-*catenin* activities within a cell (Sanders et al.,^2009^), and *Notch* signaling promotes dorsal identity of the PAs (Zuniga et al.,^2010^). Together, these findings suggest that genetic modifications of *Wnt/β-catenin* signaling activity may be involved in deploying localized epithelial signals as well as species-specific patterning of the vertebrate jaw.

## EXPERIMENTAL PROCEDURES

### Mice

All animal studies were performed according to protocols reviewed and approved by the Institutional Animal Care and Use Committee at the Children's Hospital Boston. Generation of the genetic modified mouse lines were reported previously: β-*cat*^*ex2*-*6*^ (Brault et al.,^2001^), β-*cat*^*ex3*^ (Harada et al.,^1999^), *Pitx1/Cre* transgenic mice (Olson et al.,^2006^), and the *R26R*^*lacZ*^ (Soriano,^1999^).

### Gene Expression and Histology Analyses

Whole-mount RNA in situ hybridization with digoxigenin-UTP-labeled RNA probes was performed as previously described (Guo et al.,^2011^). Histology and X-gal staining were preformed using essentially the same method as reported previously (Li et al.,^2002^,^2003^; Guo et al.,^2011^; Wang et al.,2011a). Edn1 plasmid was a gift from Dr. Hiroki Kurihara and Dr. Jeong Kyo Yoon (Sato et al.,^2008^; Jin et al.,^2011^).

### RNA Isolation and Real-Time Quantitative Reverse Transcription Polymerase Chain Reaction

Total RNA was isolated from tissue dissected from the first pharyngeal arch of E10.5 embryos (wild-type littermate controls and mutants). cDNA was synthesized using the AccuScript High Fidelity 1st strand cDNA Synthesis Kit. Real-time polymerase chain reaction (PCR) was performed as described in the manufacturer's protocol on an Applied Biosystems StepOnePlus. The mRNA levels of *Dlx5*, *Dlx6*, *Edn1*, *Endra*, and *Hand2* were normalized using β-*actin*. PCR was performed using the primers 5′-TCT CTA GGA CTG ACG CAA ACA-3′ and 5′-GTT ACA CGC CAT AGG GTC GC-3′ for *Dlx5*, 5′-AAA ACG ACA GTG ATC GAA AAC GG-3′ and 5′-AGT CTG CTG AAA GCG ATG GTT-3′ for *Dlx6*, 5′-GCA CCG GAG CTG AGA ATG G-3′ and 5′-GTG GCA GAA GTA GAC ACA CTC-3′ for *Edn1*, 5′-TAC AAG GGC GAG CTG CAT AG-3′ and 5′-GGC GTG CTG ATT TCC AAA GG-3′ for *Ednra*, 5′-GCA GGA CTC AGA GCA TCA ACA-3′ and 5′-AGG TAG GCG ATG TAT CTG GTG-3′ for *Hand2*, 5′-TCG TCG ACA ACG GCT CCG GCA TGT-3′ and 5′-CCA GCC AGG TCC AGA CGC AGG AT-3′ for β-*actin*. Statistical significance was determined by Student's *t*-test with *P* < 0.05.

### Immunohistochemistry

Cryostat sagittal sections (10 mm) of staged embryos were used for immunohistochemical analyses. Room-temperature slides were washed for 10 min in phosphate buffered saline (PBS), placed in blocking solution (0.1% Tween 20 and 5% goat serum in PBS) for 1 hr at room temperature, and then incubated with anti-β-catenin antibody (BD Transduction Laboratory) in blocking solution for 3 hr at room temperature. Sections were washed 3 times for 10 min each in PBST (PBS with 0.1% Tween 20) and incubated with Cy2-conjugated secondary antibody (Jackson ImmunoResearch, Inc.) in blocking solution for 2 hr at room temperature. These stained sections were counterstained with DAPI (4′,6-diamidine-2-phenylidole-dihydrochloride) and imagined using an Olympus SZX16 fluorescent microscope with a DP71 digital camera.

### Alcian Blue Cartilage Staining and Alizarin Red Bone Staining

The cartilage and bone staining were performed as reported (Li et al.,^2003^). Briefly, staged embryos were collected and fixed in 95% ethanol for more than 24 hr before staining for cartilage with Alcian blue (15–30 mg in 80% ethanol and 20% glacial acetic acid) for 2 days. Bones of the E16.5 embryos were stained with 75 μg/ml Alizarin red and 1% potassium hydroxide overnight. All stained embryos were cleared in glycerol before photo imaging using an Olympus M15 dissecting microscope.
